# Causes of death in a nationwide cohort of community-dwellers with Alzheimer’s disease

**DOI:** 10.1186/s12877-020-01744-z

**Published:** 2020-11-02

**Authors:** Anna-Maija Tolppanen, Heidi Taipale, Marjaana Koponen, Jari Tiihonen, Sirpa Hartikainen

**Affiliations:** 1 School of Pharmacy, University of Eastern Finland, PL1627, 70211 Kuopio, Finland; 2 Kuopio Research Centre of Geriatric Care, University of Eastern Finland, Kuopio, Finland; 3 Department of Clinical Neuroscience, Karolinska Institutet, Stockholm, Sweden; 4 Department of Forensic Psychiatry, Niuvanniemi Hospital, University of Eastern Finland, Kuopio, Finland; 5 Centre for Medicine Use and Safety, Faculty of Pharmacy and Pharmaceutical Sciences, Monash University, Parkville, Victoria Australia

**Keywords:** Alzheimer’s disease, Causes of death, Cohort, Mortality

## Abstract

**Background:**

Alzheimer’s disease (AD) is related to higher mortality but it is not entirely evident which causes of death explain this. The objective of this study was to assess the causes of death in a nationwide cohort of clinically verified AD cases and compare the causes to a matched comparison cohort without AD.

**Methods:**

Cohort of all community-dwellers with clinically verified AD residing in Finland on 31 December 2005 (*n* = 27,948) and a matched comparison cohort without AD (*n* = 27,948). Mortality (2006–2012, *n* = 30,641, 54.8%) and causes of death were obtained from register. Cause of death was ascertained with clinical examination (87.3% of deaths), forensic (8.0%) or medical autopsy (4.7%).

**Results:**

In AD cohort, the most common causes were diseases of the nervous system (49.9%), circulatory system (31.7%) and neoplasms (7.7%), while diseases of circulatory system (53.5%), neoplasms (19.1%) and mental and behavioral disorders (7.3%) contributed for majority of deaths in the comparison cohort. There were no sex-wise differences. People with AD were over 20 times more likely to die due to diseases of the nervous system (OR, 95% CI 22.06, 19.87–24.25) than the comparison cohort, while other causes, e.g., diseases of the circulatory system (0.40, 0.38–0.42), neoplasms (0.35, 0.33–0.38), mental and behavioral disorders (0.27, 0.24–0.30) and external causes of morbidity and mortality (0.72, 0.62–0.81) were less common in the AD cohort.

**Conclusions:**

Although half of the people with AD died due to diseases of the nervous system, cancers and especially cardio/cerebrovascular diseases were still important contributors to the overall mortality among them. This should be acknowledged when planning their terminal care.

**Supplementary Information:**

The online version contains supplementary material available at 10.1186/s12877-020-01744-z.

## Background

Persons with Alzheimer’s disease, the most common form of dementia, have higher mortality in comparison to matched population without dementia [1–4]. The increase in mortality has been implied to be larger in men [5], and pneumonia and cardiovascular diseases are suggested as the main contributors for the increased mortality [6–13]. Most [7–11] of these studies have been autopsy series, and have thus included a selected sample of persons with Alzheimer’s disease (AD). As only few population-based cohort studies on the causes of death have been conducted [6, 12, 13], it is not entirely evident which causes of death explain the difference in mortality. A recent study, based on the Swedish dementia register showed that circulatory causes were the most common underlying cause of death in persons with dementia (37% of deaths), followed closely by dementia (30% of deaths) [13].

We investigated the causes of death in a nationwide sample of community-dwelling persons with clinically verified AD diagnosis and compared them to a matched comparison cohort without AD. We also assessed whether the causes of death were different among those who underwent medical or forensic autopsy. Since registers, including the causes of death register are sometimes used to ascertain dementia/AD status in epidemiological studies, we assessed which proportion of the clinically verified AD cases would be identified with the causes of death register. Previous studies from England and Wales [14] and Sweden [13] have shown that although recording dementia on death certificate has improved over time [14], it is still an underestimate.

## Methods

### Study population

The Medication and Alzheimer’s Disease 2005 (MEDALZ-2005) study cohort includes all community-dwelling persons with a verified diagnosis of Alzheimer’s disease residing in Finland on 31 December 2005 (*n* = 28,093) and a single age-, sex- and region of residence- matched comparison person for each individual with AD (n of comparison cohort = 28,093, *N* = 56,186) [15]. The age range of the cohort was 42–101 years (mean 79.9 (SD 6.8) years) and 38,086 (67.8%) of the sample were women. Each resident of Finland is assigned a personal identification number which was used to obtain data on mortality and causes of death between Jan 1, 2006 and Dec 31, 2012 from the Causes of death register, maintained by the Statistics Finland. Data on comorbidities were obtained from national registers as described in Supplementary Table 1.

### Diagnosis of Alzheimer’s disease

Persons with AD were identified from the Finnish Special Reimbursement Register maintained by the Social Insurance Institution of Finland (SII) as described in detail previously [15]. Briefly, the specific criterion for a verified AD diagnosis is 1) symptoms consistent with mild or moderate AD, 2) a decrease in social capacity over a period of at least 3 months, 3) a computer tomography/magnetic resonance imaging scan, 4) exclusion of other reasons for symptoms (for example vascular or frontotemporal dementia, hyperparathyreoidism, hypothyreoidism or severe depletion of vitamin B12), and 5) confirmation of the diagnosis by a registered neurologist or geriatrician. The AD diagnosis was based on the NINCDS-ADRDA and DSM-IV criteria for Alzheimer’s disease [16, 17]. Persons with some findings related to other dementive disorder (e.g. a few lacunar infarcts) were included if the symptoms were mainly caused by AD. The comparison persons were identified from the register that contains all residents of Finland who are entitled to benefits by the SII, i.e. all citizens and residents living in Finland for at least 2 years.

### Causes of death

The Causes of death register is compiled from death certificate data. It covers persons who have died in Finland or abroad during the calendar year and who at the time of death were domiciled in Finland. Underlying cause of death, direct cause, intervening causes and up to four contributing causes are registered.

Death certificates are issued by physicians and if an autopsy is required, the death certificate is issued by a medicolegal officer after completion of the autopsy. Death certificates are submitted to the regional unit of the National Institute for Health and Welfare of the region where the deceased resided. Provincial medical officer confirms the certificates before they are sent on to Statistics Finland for registration. Forensic autopsies were performed for 21%, and medical autopsy for 6.7% of all deaths in Finland 2011 [18].

In this study, the underlying and direct causes of death were classified to main classes of according to ICD-10 classification system (Table 1). Underlying cause is the disease that initiated the series of illnesses leading directly to death, or the circumstances connected with an accident/act of violence which caused the injury/poisoning leading to death. Direct cause refers to the disease, failure or injury whose symptoms caused the death.

**Table 1:** Underlying and Direct Causes of Death in the Comparison (No AD) and AD Cohorts of the MEDALZ-2005 Study

Category	ICD-10 code	No AD, *n* = 10,171	AD, *n* = 20,470	OR (95%CI
Diseases of the circulatory system	I*	5441 (53.5)	6484 (31.7)	0.40 (0.38, 0.42)
Diseases of the nervous system	G*	440 (4.3)	10,218 (50.0)	22.06 (19.87, 24.25)
Neoplasms	C*, D00-D48	1942 (19.1)	1573 (7.7)	0.35 (0.33, 0.38)
Mental and behavioral disorders	F*	741 (7.3)	425 (2.1)	0.27 (0.24, 0.30)
Diseases of the respiratory system	J*	512 (5.0)	380 (1.9)	0.36 (0.31, 0.40)
External causes of morbidity and mortality	V*, W*, X*, Y*	356 (3.5)	518 (2.5)	0.72 (0.62, 0.81)
Diseases of the digestive system	K*	308 (3.0)	423 (2.1)	0.68 (0.58, 0.78)
Certain infectious and parasitic diseases	A*, B*	89 (0.9)	96 (0.5)	0.53 (0.40, 0.71)
Diseases of the genitourinary system	N*	132 (1.3)	156 (0.8)	0.58 (0.46, 0.74)
Endocrine, nutritional and metabolic diseases	E*	115 (1.1)	116 (0.6)	0.50 (0.38, 0.65)
Diseases of the musculoskeletal system and connective tissue	M*	49 (0.5)	21 (0.1)	0.21 (0.13, 0.35)
Diseases of the blood and blood-forming organs and certain disorders involving the immune mechanism	D50-D89	10 (0.1)	11 (0.1)	0.55 (0.23, 1.29)
Symptoms, signs and abnormal clinical and laboratory findings, not elsewhere classified	R*	10 (0.1)	17 (0.1)	0.84 (0.39, 1.85)
Congenital malformations, deformations and chromosomal abnormalities	Q*	8 (0.1)	13 (0.1)	0.81 (0.33, 1.95)
Diseases of the skin and subcutaneous tissue	L*	9 (0.1)	4 (< 0.1)	0.22 (0.07, 0.72)
Diseases of the eye, adnexa, ear and mastoid process	H*	2 < (0.1)	0 (0.0)	N.A

**Table 2:** Underlying and Direct Causes of Death among People with AD Stratified by Age at AD Diagnosis

Category	ICD-10 code	< 65 years *n* = 409	≥65 years *n* = 20,061	OR (95%CI
Diseases of the circulatory system	I*	48 (11.7)	6436 (32.1)	3.55 (2.47–4.62)
Diseases of the nervous system	G*	293 (71.6)	9925 (49.5)	0.38 (0.30–0.47)
Neoplasms	C*, D00-D48	18 (4.4)	1555 (7.8)	1.82 (0.96–2.69)
Mental and behavioral disorders	F*	12 (2.9)	413 (2.1)	0.69 (0.29–1.10)
Diseases of the respiratory system	J*	5 (1.2)	375 (1.9)	1.54 (0.17–2.90)
External causes of morbidity and mortality	V*, W*, X*, Y*	10 (2.4)	508 (2.5)	1.03 (0.38–1.69)
Diseases of the digestive system	K*	6 (1.5)	417 (2.1)	1.42 (0.27–2.58)
Certain infectious and parasitic diseases	A*, B*	0 (0)	96 (0.5)	N. A
Diseases of the genitourinary system	N*	1 (0.2)	155 (0.8)	3.17 (0.01–9.41)
Endocrine, nutritional and metabolic diseases	E*	4 (1.0)	112 (0.6)	0.57 (0.00–1.14)
Diseases of the musculoskeletal system and connective tissue	M*	1 (0.2)	20 (0.1)	0.41 (0.00–1.22)
Diseases of the blood and blood-forming organs and certain disorders involving the immune mechanism	D50-D89	0 (0)	11 (0.1)	N. A
Symptoms, signs and abnormal clinical and laboratory findings, not elsewhere classified	R*	1 (0.2)	16 (0.1)	0.33 (0.01–0.98)
Congenital malformations, deformations and chromosomal abnormalities	Q*	9 (2.2)	4 (< 0.1)	0.01 (0.00–0.02)
Diseases of the skin and subcutaneous tissue	L*	0 (0)	4 (< 0.01)	N. A

### Statistical analyses

Altogether 145 persons in the comparison cohort had been temporarily entitled to reimbursed AD medication before 2006 and they, together with their matched pairs, were excluded from the study. Of the remaining 55,896 participants, 3415 converted to AD during the follow-up and deaths were ascertained to AD cohort (according to AD status at the time of death). These people had no matched comparison person without AD, and we also performed sensitivity analyses excluding these 3415 pairs but this did not have a significant effect on the results (data not shown). Differences on the causes of death were assessed with risk ratios, derived from logistic regression. Possible sex differences were investigated by including a sex*AD interaction term in the model. Age-stratified analyses, with 65 years as a cutoff were performed in people with AD to assess possible differences between those with early and late-onset AD. Associations between comorbidities and mortality were investigated with logistics regression.

We assessed how often Alzheimer’s Disease (ICD-10 code G30) or dementia (F00, F01, F02, F03, G30, F051, F107, F117, F147, F167, F187 and F197) were listed as underlying, direct or intervening cause of death or any cause of death and whether AD was more often mentioned in the death certificate for those with longer duration of disease using logistic regression. Duration of disease was defined as time since clinically verified AD diagnosis at the time of death. Likelihood ratio tests demonstrated nonlinear association between AD duration and likelihood of having AD or dementia listed as cause of death. Therefore, we do not present OR per one-year increase in disease duration.

## Results

### Sample characteristics

Altogether 30,641 cohort members (20,470 with AD, 10,171 without AD) died during the follow-up. The median length of the follow-up was 6.3 years (range 1 day to 7 years). Consistent with our previous results on survival from the same cohort with shorter follow-up (4.8 years) [1], men were approximately 50% more likely to die than women and the sex difference was more pronounced in the AD cohort (HR, 95% CI 1.59, 1.55–1.64) than in the non-AD group (HR 1.47, 1.41–1.53). People with AD were 1.8 years younger when they died (average age at death 84.4 and 86.2 years for AD and non-AD groups, respectively). In both AD and comparison cohorts, all baseline comorbidities (cardiovascular disease, diabetes, stroke, asthma/chronic obstructive pulmonary disease, hip fracture, rheumatoid arthritis and cancer) were associated with mortality (Supplementary Table 2).

Information on any cause of death was not available for 14 participants in the AD cohort and 8 in the comparison cohort (0.07 and 0.07%, respectively). These persons were excluded from the analysis. Underlying or direct cause of death was listed for 30,619 individuals (97.3%), ascertained with clinical examination for 89.7% of the AD cohort (*n* = 18,357) and 82.1% (*n* = 8352) of the comparison cohort. Of the AD cohort, 6.0% (*n* = 1234) underwent forensic autopsy and 4.2% (*n* = 851) medical autopsy while 11.9% (*n* = 1210) and 5.8% (*n* = 594) of the comparison cohort underwent forensic and medical autopsy, respectively.

### Causes of death

In the AD cohort, the most common main/direct causes of death were diseases of the nervous system (49.9%), circulatory system (31.7%) and neoplasms (7.7%), accounting for nearly 90% of deaths in this cohort (Table 1). In the comparison cohort, the most common causes of death were diseases of circulatory system (53.5%), neoplasms (19.1%), mental and behavioral disorders (7.3%) and diseases of the nervous system (4.3%), accounting for 84.2% of deaths. The frequency of other causes was < 5% in both groups and the absolute differences between groups were small for these less common causes. People with AD were less likely to die due to diseases of the circulatory, respiratory, digestive, genitourinary or musculoskeletal system, neoplasms, external causes of morbidity and mortality or mental and behavioral disorders and as expected, over 22 times more likely to die due to diseases of the nervous system.

More detailed diagnoses are listed in Supplementary Table 3. Ischemic heart disease and stroke were the most common circulatory diseases in both cohorts and lung cancer was the most common cancer diagnosis. Of diseases of the nervous system, Alzheimer’s disease was the most common followed by Parkinson’s disease in both cohorts. Mental and behavioral disorder diagnoses were mainly dementia diagnoses in both cohorts.

In the age at diagnosis-stratified analysis of AD cases, diseases of circulatory system were more commonly recorded as the main or direct cause of death among those with later onset (Table 2). Diseases of the nervous system were more common among those with earlier onset. Congenital malformations, deformations and chromosomal abnormalities, together with abnormal laboratory findings were also more commonly listed as the main cause of death for those with younger age at AD diagnosis, but the number of these cases was fairly low.

In the AD cohort, the distribution of most causes was similar in both sexes (Table 3). Negative association between AD and deaths due to diseases of the circulatory system and mental and behavioral disorders and the positive association between AD and diseases of nervous system were stronger in men than in women.

**Table 3:** Underlying and Direct Causes of Death among Men and Women

Category	Men *N* = 10,934		Women *N* = 19,707		*P* for sex difference
	No AD *n* = 3654	AD *n* = 7280	No AD *n* = 6517	AD *n* = 13,190	
Diseases of the circulatory system	1842 (50.4)	2202 (30.2)	3599 (55.2)	4282 (32.5)	0.078
Diseases of the nervous system	123 (3.4)	3541 (48.6)	317 (4.9)	6677 (50.6)	0.006
Neoplasms	830 (22.7)	667 (9.2)	1112 (17.1)	906 (6.9)	0.56
Mental and behavioral disorders	191 (5.2)	152 (2.1)	550 (8.4)	273 (2.1)	< 0.001
Diseases of the respiratory system	280 (7.7)	228 (3.1)	232 (3.6)	152 (1.2)	0.13
External causes of morbidity and mortality	141 (3.9)	197 (2.7)	215 (3.3)	321 (2.4)	0.71
Diseases of the digestive system	101 (2.8)	136 (1.9)	207 (3.2)	287 (2.2)	0.94
Certain infectious and parasitic diseases	30 (0.8)	37 (0.5)	59 (0.9)	59 (0.4)	0.46
Diseases of the genitourinary system	46 (1.3)	45 (0.6)	86 (1.3)	111 (0.8)	0.31
Endocrine, nutritional and metabolic diseases	41 (1.1)	44 (0.6)	74 (1.1)	72 (0.5)	0.67
Diseases of the musculoskeletal system and connective tissue	12 (0.3)	4 (0.1)	37 (0.6)	17 (0.1)	0.64
Diseases of the blood and blood-forming organs and certain disorders involving the immune mechanism	3 (0.1)	5 (0.1)	7 (0.1)	6 (< 0.1)	0.46
Symptoms, signs and abnormal clinical and laboratory findings, not elsewhere classified	6 (0.2)	7 (0.1)	4 (0.1)	10 (0.1)	0.36
Congenital malformations, deformations and chromosomal abnormalities	1 (< 0.1)	5 (0.1)	7 (0.1)	8 (0.1)	0.22
Diseases of the skin and subcutaneous tissue	4 (0.1)	2 (< 0.1)	5 (0.1)	2 (< 0.1)	0.84
Diseases of the eye, adnexa, ear and mastoid process	1 (< 0.1)	0 (0.0)	1 (< 0.1)	0 (0.0)	N.A

### Causes of death in autopsied participants

Causes of death for autopsied participants are listed in Table 4. As with all deaths (Table 1), deaths due to circulatory causes and neoplasms were less common and deaths due to diseases of the nervous system more common in the AD cohort in the autopsied subsample. Diseases of the circulatory system were the most common autopsy-confirmed reason for death (> 50%) in both cohorts, regardless of autopsy type (medical or forensic). In the subgroup autopsied for medical reasons, other common autopsy-confirmed causes of death were neoplasms and diseases of the digestive system (≥10.0% in both cohorts) and diseases of the nervous system (17.7% in the AD cohort). Approximately one fourth of the deaths in the AD cohort and one fifth of the deaths in the comparison cohort confirmed with forensic autopsy were due to external causes.

**Table 4:** Underlying and Direct Causes of Death in Autopsied Participants of the MEDALZ-2005 Study

Category	All autopsies (*N* = 3889)			Medical autopsies (*N* = 1445)			Forensic autopsies (*N* = 2444)		
	No AD(*n* = 1804)	AD (*n* = 2085)	OR (95%CI	No AD (*n* = 594)	AD (*n* = 851)	OR (95%CI	No AD (*n* = 1210)	AD (*n* = 1234)	OR (95%CI
Diseases of the circulatory system	1131 (62.7)	1099 (52.7)	0.66 (0.58, 0.75)	361 (60.8)	447 (52.5)	0.71 (0.58, 0.88)	770 (63.6)	652 (52.8)	0.64 (0.54, 0.75)
External causes of morbidity and mortality	257 (14.2)	331 (15.9)	1.14 (0.95, 1.36)	0 (0.0)	5 (0.6)	N.A	257 (21.2)	326 (26.4)	1.33 (1.10, 1.61)
Neoplasms	171 (9.5)	150 (7.2)	0.74 (0.59, 0.93)	109 (18.4)	102 (12.0)	0.61 (0.45, 0.81)	62 (5.1)	48 (3.9)	0.75 (0.51, 1.10)
Diseases of the digestive system	122 (6.8)	148 (7.1)	1.05 (0.82, 1.35)	61 (10.3)	95 (11.2)	1.10 (0.78, 1.54)	61 (5.0)	53 (4.3)	0.85 (0.58, 1.23)
Diseases of the nervous system	7 (0.4)	259 (12.4)	36.41 (17.14, 77.36)	4 (0.7)	152 (17.9)	32.07 (11.81, 87.08)	3 (0.2)	107 (8.7)	38.20 (12.09, 120.66)
Diseases of the respiratory system	60 (3.3)	51 (2.4)	0.73 (0.50, 1.06)	23 (3.9)	21 (2.5)	0.63 (0.34, 1.15)	37 (3.1)	30 (2.4)	0.79 (0.49, 1.29)
Endocrine, nutritional and metabolic diseases	12 (0.7)	10 (0.5)	0.72 (0.31, 1.67)	7 (1.2)	5 (0.6)	0.50 (0.16, 1.56)	5 (0.4)	5 (0.4)	0.98 (0.28, 3.40)
Diseases of the genitourinary system	11 (0.6)	11 (0.5)	0.86 (0.37, 2.00)	8 (1.3)	9 (1.1)	0.78 (0.30, 2.04)	3 (0.2)	2 (0.2)	0.65 (0.11, 3.92)
Certain infectious and parasitic diseases	12 (0.7)	8 (0.4)	0.58 (0.24, 1.41)	10 (1.7)	6 (0.7)	0.41 (0.15, 1.15)	2 (0.2)	2 (0.2)	0.98 (0.14, 6.97)
Mental and behavioral disorders	9 (0.5)	11 (0.5)	1.06 (0.44, 2.56)	7 (1.2)	9 (1.1)	0.90 (0.33, 2.42)	2 (0.2)	2 (0.2)	0.98 (0.14, 6.97)
Musculoskeletal system/ connective tissue diseases	7 (0.4)	1 (< 0.1)	0.12 (0.02, 1.00)	3 (0.5)	0 (0.0)	NA	4 (0.3)	1 (0.1)	0.24 (0.03, 2.19)
Symptoms, signs, abnormal clinical/laboratory findings	3 (0.2)	4 (0.2)	1.15 (0.26, 5.16)	0 (0.0)	0 (0.0)	NA	3 (0.2)	4 (0.3)	1.31 (0.30, 5.86)
Congenital malformations, deformations and chromosomal abnormalities	2 (0.1)	1 (< 0.1)	0.43 (0.04, 4.77)	1 (0.2)	0 (0.0)	NA	1 (0.1)	1 (0.1)	0.98 (0.06, 15.69)
Diseases of the blood and blood-forming organs and certain disorders involving the immune mechanism	0 (0.0)	1 (< 0.1)	NA	0 (0.0)	0 (0.0)	NA	0 (0.0)	1 (0.1)	N.A

### Alzheimer’s disease as a cause of death

**Fig. 1:**
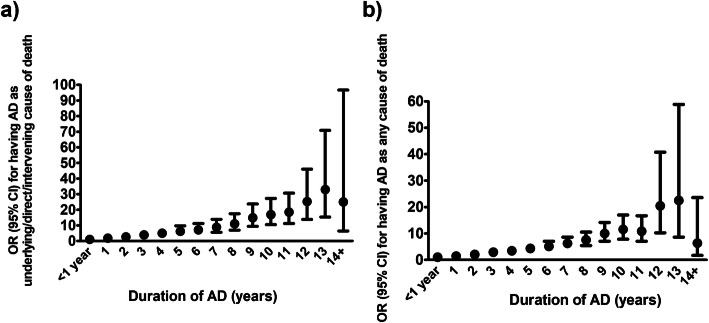
Odds for including AD as **a** an underlying direct or intervening cause of death or **b** any cause of death according to duration of AD

Alzheimer’s disease was included as an underlying, direct or intervening cause of death for 48.3% (*n* = 9880) of people in the AD cohort and 2.8% (*n* = 282) people in the comparison cohort. Altogether 71.0% (*n* = 14,537) of the AD cohort and 4.1% (*n* = 413) of the matched comparison cohort had AD listed as any cause of death (including also contributory causes). Fifty-one percent (*n* = 10,407) of the AD group and 10.2% (*n* = 1042) of the comparison cohort had any form of dementia listed as an underlying, direct or intervening cause of death and 75.3% (*n* = 15,404) of the death certificates of AD cases and 15.6% (*n* = 1589) of the comparison group had any mention of dementia. Of these 1589 persons, 42.4% had unspecified dementia, 29.0% had vascular dementia and 26.0% had Alzheimer’s disease. The remaining 2.6% had other forms of dementia. The likelihood for having AD as the underlying, direct or intervening cause of death increased nonlinearly with the duration of disease until (Fig. 1a, Supplementary Table 4). Similar association was observed between any mention of AD in the death certificate and years since AD diagnosis (Fig. 1b, Supplementary Table 4).

When the analyses were restricted to those deaths that were not due to dementia (i.e. did not have dementia as underlying, direct or underlying cause of death), diseases of the circulatory system and neoplasms remained as the most common underlying/direct cause of death. Diseases of the circulatory system accounted for 62.1% deaths in the whole cohort and 59.6 and 64.4% of deaths in the non-AD and AD cohorts, respectively (Table 5). In comparison to results presented in Table 1, the proportion of deaths due to neoplasms increased in the AD cohort and the proportion of deaths due to diseases of the nervous system decreased significantly.

**Table 5:** Underlying and Direct Causes of Death in the Comparison (No AD) and AD Cohorts of the MEDALZ-2005 Study after Excluding Deaths with Dementia as the Underlying, Direct or Intermediate Cause of Death

Category	ICD-10 code	No AD, *n* = 9129	AD, *n* = 10,063	OR (95%CI)
Diseases of the circulatory system	I*	5438 (59.6)	6481 (64.4)	1.23 (1.16, 1.30)
Diseases of the nervous system	G*	124 (1.4)	234 (2.3)	1.73 (1.39, 2.16)
Neoplasms	C*, D00-D48	1942 (21.3)	1573 (15.6)	0.69 (0.64, 0.74)
Mental and behavioral disorders	F*	18 (0.2)	5 (< 0.1)	0.25 (0.09, 0.68)
Diseases of the respiratory system	J*	512 (5.6)	380 (3.8)	0.66 (0.58, 0.74)
External causes of morbidity and mortality	V*, W*, X*, Y*	356 (3.9)	518 (5.1)	1.34 (1.17, 1.54)
Diseases of the digestive system	K*	308 (3.4)	423 (4.2)	1.26 (1.08, 1.46)
Certain infectious and parasitic diseases	A*, B*	89 (1.0)	96 (1.0)	0.98 (0.73, 1.31)
Diseases of the genitourinary system	N*	132 (1.4)	156 (1.6)	1.07 (0.85, 1.36)
Endocrine, nutritional and metabolic diseases	E*	115 (1.3)	116 (1.2)	0.91 (0.71, 1.19)
Diseases of the musculoskeletal system and connective tissue	M*	49 (0.5)	21 (0.2)	0.39 (0.23, 0.64)
Diseases of the blood and blood-forming organs and certain disorders involving the immune mechanism	D50-D89	10 (0.1)	11 (0.1)	1.00 (0.42, 2.35)
Symptoms, signs and abnormal clinical and laboratory findings, not elsewhere classified	R*	10 (0.1)	17 (0.2)	1.54 (0.71, 3.37)
Congenital malformations, deformations and chromosomal abnormalities	Q*	8 (0.1)	13 (0.1)	1.48 (0.61, 3.56)
Diseases of the skin and subcutaneous tissue	L*	9 (0.1)	4 (< 0.1)	0.40 (0.12, 1.31)
Diseases of the eye, adnexa, ear and mastoid process	H*	< 2 (0.1)	0 (0.0)	N.A

## Discussion

Diseases of the nervous system, including AD, were the most common underlying or direct cause of death in our nationwide cohort of community-dwelling persons with AD, accounting for 50% of deaths. This is expected, as AD per se is related to higher mortality [1–5]. In our study, diseases of the circulatory system and neoplasms were among the leading causes in those with and without AD, but they were more common in the comparison cohort without AD. The differences in causes of death by age at AD diagnosis were not surprising as older persons have more cardiovascular comorbidities.

AD was listed as a cause of death for 4.1% of the comparison group. As our AD diagnoses were obtained from the Special reimbursement register (i.e. patients with AD who were eligible for reimbursed AD medication), these comparison persons may have been persons with AD who were not deemed to benefit from anti-dementia medication, may have had contraindications for these medicines or had not received the decision for reimbursed medication before their death.

Since most of the previous studies are autopsy series, our results are not directly comparable with them. Death certificates are meant to reflect the relevant information regarding cause of death, but the degree of precision may vary within and between institutions, as well as over time. In a previous validation study of 58 dementia cases, the agreement between death certificate and autopsy was 53% and especially the contribution of bronchopneumonia was underestimated [11]. Although the proportion of deaths due to respiratory conditions, including bronchopneumonia, was much lower in our study than in the previous ones that have reported estimates for persons with AD [7–9, 11–13], the proportion of persons with diseases of circulatory system was very similar to that reported in these studies. In 2005, the Finnish classification of bronchopneumonia deaths was changed so that bronchopneumonia was less often accepted as a main cause of death for those with long-term illness [18]. This change, deriving from ICD-9 to ICD-10 transition, may explain the lower proportion of deaths due to bronchopneumonia in our study (follow-up 2006–2013) because all deaths in the previous studies occurred earlier [7–9, 11, 12]. However, in one Swedish autopsy study with higher proportion of bronchopneumonia deaths, the causes of death were ascertained according to ICD-10 [8].

The differences between ours and previous results can also partially be due to difficulties in ascertaining the cause of death without autopsy. However, it should be noted that our sample represents people with AD who were community-dwelling in the beginning of the study and thus they may be very different to those people with AD who undergo a medical autopsy. Some of the previous cohort studies have also included cases from a long period of time [6, 8]. This, together with changes in life expectancy and treatment of people with AD and temporal changes in causes of death [18, 19], means that the differences may also reflect true differences in the contributing causes. Further, there may be some variation in the causes of death depending on level of cognitive impairment [20], but this kind of data were not available for our study.

One of the strengths of our study is the availability of matched controls. Due to the carefully maintained national registers of Finland, we were able to obtain a nationwide sample of all clinically verified AD cases at certain time point and obtain accurate data on time of death, as well as the information of death certificates for all deceased individuals. We were also able to assess whether the causes of death were different among those who underwent forensic or medical autopsy.

Our data is not in agreement with the suggestion that additional causes of death might not be considered if the deceased was already diagnosed with AD or other form of dementia as approximately 50% of deaths in the AD cohort were assigned to causes other than AD or dementia. AD was mentioned as a cause of death for 70% of clinically verified AD cases, a proportion comparable to a previous study based on Swedish Dementia register [13]. This has implications for using the causes of death register for AD status ascertainment in epidemiological studies. The lack of mention of dementia/AD in all certificates of the AD group does not necessarily reflect incompetent or incomplete certification, because dementia/AD may not have contributed to death in all cases. In a previous study, dementia was less often disclosed in the death certificate when the duration of disease was less than 5 years [12]. This is in line with our findings on the positive association between AD duration and inclusion of AD in the death certificate.

## Conclusions

Although half of the people with AD in our nationwide cohort died of diseases of the nervous system, cancers and especially cardio- and cerebrovascular diseases were still important contributors to the overall mortality among them. This finding was confirmed in the autopsied subsample. Although men with AD had shorter survival in comparison to women, there were no important sex differences in the causes of death. This knowledge about the causes death is valuable for planning the terminal care of patients with AD.

## Supplementary Information

**Additional file 1: Supplementary Table 1.** Definition of Comorbidities. **Supplementary Table 2.** Distribution of Comorbidities in the AD and Comparison Cohorts According to Mortality. **Supplementary Table 3.** Frequency of Main and Indirect Causes of Death in the AD and Comparison Cohorts of the MEDALZ-2005 Study. Only Those Causes With Frequency > 0.5% in Either of the Cohorts Are Listed. **Supplementary Table 4.** Odds Ratios for Having AD Listed as Main or Any Cause of Death According to Time since AD Diagnosis.

## Data Availability

The data that support the findings of this study are available from the corresponding author but restrictions apply to the availability of these data, and so the data are not publicly available. Data are however available from the authors upon reasonable request and with permission of the register maintainers.

## Abbreviations

AD: Alzheimer’s disease
MEDALZ-2005: The Medication and Alzheimer’s Disease 2005 study
SII: Social Insurance Institution of Finland
