# Supplementary material for: Causes of death in a nationwide cohort of community-dwellers with Alzheimer’s disease
Source: BMC Geriatr. 2020 Nov 2;20:441. doi: 10.1186/s12877-020-01744-z (PMC7607696; doi:10.1186/s12877-020-01744-z)
Supplement: Supplementary file 1 — Additional file 1: Supplementary Table 1. Definition of Comorbidities. Supplementary Table 2. Distribution of Comorbidities in the AD and Comparison Cohorts According to Mortality. Supplementary Table 3. Frequency of Main and Indirect Causes of Death in the AD and Comparison Cohorts of the MEDALZ-2005 Study. Only Those Causes With Frequency > 0.5% in Either of the Cohorts Are Listed. Supplementary Table 4. Odds Ratios for Having AD Listed as Main or Any Cause of Death According to Time since AD Diagnosis. [file 12877_2020_1744_MOESM1_ESM.docx]

Supplementary Table 1 Definition of Comorbidities.

| Comorbidity | Data sources & coding |
| --- | --- |
| Cardiovascular disease | Special reimbursement register codes 201 (heart failure), 205 (hypertension), 206, 213, 280 (coronary artery disease) |
| Diabetes | Prescription register: ATC code A10 excluding A10BX01(guar gum)  Special reimbursement register code 103 |
| Stroke | Care register for health care ICD-10 I60-I64, I69 |
| Asthma/chronic obstructive pulmonary disease | Special reimbursement register code 203  Care register for health care ICD-10 J44-J46 |
| Hip fracture | Care register for health care ICD-10 S72.0-S72.2 |
| Rheumatoid arthritis | Special reimbursement register code 202 |
| Cancer | Care register for health care ICD-10 C  Prescription register: ATC codes L01 (antineoplastic agents), L02 (endocrine therapy), L03AA (colony stimulating factors), L03AB01 (interferon alpha natural), L03AB04 (interferon alpha-2a), L03AB05 (interferon alpha-2b), L03AC (interleukins), L03AX (other immunostimulants, excluding L03AX13, glatiramer acetate), L04AA10 (sirolimus), L04AA18 (everolimus), L04AA34 (alemtuzumab), L04AX02 (thalidomide), and L04AX03 or L01BA01 (methotrexate, excluding persons with a Special Reimbursement for rheumatoid arthritis) |

Supplementary Table 2 Distribution of Comorbidities in the AD and Comparison Cohorts According to Mortality

| Comorbidity | AD cohort n (%) | | | Comparison cohort n (%) | | |
| --- | --- | --- | --- | --- | --- | --- |
|  | Alive, n=10,893 | Dead, n=20,470 | P | Alive, n=14,362 | Dead, n=10,171 | P |
| Cardiovascular disease | 4,576 (42.0) | 10,442 (51.0) | <0.001 | 5,992 (41.7) | 5,821 (57.2) | <0.001 |
| Diabetes | 1,947 (17.9) | 4,843 (23.7) | <0.001 | 913 (6.4) | 1,273 (12.5) | <0.001 |
| Stroke | 434 (4.0) | 1,253 (6.1) | <0.001 | 392 (2.7) | 938 (9.2) | <0.001 |
| Asthma/Chronic obstructive pulmonary disease | 830 (7.6) | 1,753 (8.6) | 0.004 | 1,069 (7.4) | 1,099 (10.8) | <0.001 |
| Hip fracture | 192 (1.8) | 707 (3.5) | <0.001 | 145 (1.0) | 443 (4.4) | <0.001 |
| Rheumatoid arthritis | 330 (3.0) | 888 (4.3 | <0.001 | 442 (3.1) | 524 (5.2) | <0.001 |
| Active cancer treatment | 916 (8.4) | 2,380 (11.6) | <0.001 | 690 (4.8) | 901 (8.9) | <0.001 |

Supplementary Table 3 Frequency of Main and Indirect Causes of Death in the AD and Comparison Cohorts of the MEDALZ-2005 Study. Only Those Causes With Frequency >0.5% in Either of the Cohorts Are Listed.

| ICD-10 code | Disease | Comparison cohort, n (%) | AD cohort, n (%) |
| --- | --- | --- | --- |
| I25 | Chronic ischemic heart disease | 1925 (18.9) | 2008 (9.8) |
| I21 | Acute myocardial infarction | 1256 (12.3) | 1707 (8.3) |
| I63 | Cerebral infarction | 607 (6.0) | 961 (4.7) |
| I61 | Nontraumatic intracerebral hemorrhage | 159 (1.6) | 232 (1.1) |
| I70 | Atherosclerosis | 105 (1.0) | 182 (0.9) |
| I69 | Sequelae of cerebrovascular disease | 272 (2.7) | 180 (0.9) |
| I11 | Hypertensive heart disease | 197 (1.9) | 175 (0.9) |
| I50 | Heart failure | 153 (1.5) | 150 (0.7) |
| I48 | Atrial fibrillation and flutter | 105 (1.0) | 125 (0.6) |
| I35 | Nonrheumatic aortic valve disorders | 132 (1.3) | 87 (0.4) |
| I64 | Stroke, not specified as haemorrhage or infarction | 37 (0.4) | 116 (0.6) |
| I71 | Aortic aneurysm and dissection | 103 (1.0) | 89 (0.4) |
| G30 | Alzheimer's disease | 282 (2.8) | 9880 (48.3) |
| G20 | Parkinson's disease | 100 (1.0) | 158 (0.8) |
| G31 | Other degenerative diseases of nervous system, not elsewhere classified | 22 (0.2) | 130 (0.6) |
| C34 | Malignant neoplasm of bronchus and lung | 283 (2.8) | 173 (0.8) |
| C61 | Malignant neoplasm of prostate | 174 (1.7) | 141 (0.7) |
| C25 | Malignant neoplasm of pancreas | 166 (1.6) | 136 (0.7) |
| C18 | Malignant neoplasm of colon | 134 (1.3) | 110 (0.5) |
| C50 | Malignant neoplasm of breast | 118 (1.2) | 107 (0.5) |
| C16 | Malignant neoplasm of stomach | 70 (0.7) | 71 (0.3) |
| C80 | Malignant neoplasm without specification of site | 54 (0.5) | 65 (0.3) |
| C22 | Malignant neoplasm of liver and intrahepatic bile ducts | 74 (0.7) | 64 (0.3) |
| C20 | Malignant neoplasm of rectum | 61 (0.6) | 59 (0.3) |
| C67 | Malignant neoplasm of bladder | 67 (0.7) | 50 (0.2) |
| C64 | Malignant neoplasm of kidney, except renal pelvis | 53 (0.5) | 45 (0.2) |
| C56 | Malignant neoplasm of ovary | 58 (0.6) | 34 (0.2) |
| C83 | Non-follicular lymphoma | 55 (0.5) | 36 (0.2) |
| F03 | Unspecified dementia | 413 (4.1) | 243 (1.2) |
| F01 | Vascular dementia | 306 (3.0) | 175 (0.9) |
| J44 | Other chronic obstructive pulmonary disease | 202 (2.0) | 181 (0.9) |
| J18 | Pneumonia, unspecified organism | 119 (1.2) | 64 (0.3) |
| J84 | Other interstitial pulmonary diseases | 69 (0.7) | 34 (0.2) |
| W01 | Fall on same level from slipping, tripping and stumbling | 170 (1.7) | 294 (1.4) |
| N10 | Acute pyelonephritis | 51 (0.5) | 52 (0.3) |
| E11 | Type 2 diabetes mellitus | 100 (1.0) | 102 (0.5) |

Supplementary Table 4 Odds Ratios for Having AD Listed as Main or Any Cause of Death According to Time since AD Diagnosis.

| Time since AD diagnosis (years) | AD as main cause of death | | | AD as any cause of death | | |
| --- | --- | --- | --- | --- | --- | --- |
|  | n (%) with other main cause | n (%) with AD as main cause | OR (95% CI) | n (%) with no mention of AD | n (%) with AD as any cause | OR (95% CI) |
| <1 | 150 (1.4) | 22 (0.2) | 1.00 (reference) | 109 (1.8) | 63 (0.4) | 1.00 (reference) |
| 1 | 666 (6.3) | 174 (1.8) | 1.78 (1.10-2.87) | 455 (7.7) | 385 (2.6) | 1.46 (1.04-2.05) |
| 2 | 1215 (11.5) | 478 (4.8) | 2.68 (1.69-4.25) | 778 (13.1) | 915 (6.3) | 2.03 (1.47-2.82) |
| 3 | 1521 (14.4) | 872 (8.8) | 3.91 (2.48-6.16) | 894 (15.1) | 1499 (10.3) | 2.90 (2.10-4.00) |
| 4 | 1606 (15.2) | 1174 (11.9) | 4.98 (3.17-7.85) | 927 (15.6) | 1853 (12.7) | 3.46 (2.51-4.76) |
| 5 | 1523 (14.4) | 1386 (14.0) | 6.20 (3.94-9.76) | 826 (13.9) | 2083 (14.3) | 4.36 (3.17-6.01) |
| 6 | 1370 (12.9) | 1436 (14.5) | 7.15 (4.54-11.25) | 708 (11.9) | 2098 (14.4) | 5.13 (3.72-7.07) |
| 7 | 1120 (10.6) | 1452 (14.7) | 8.84 (5.61-13.92) | 557 (9.4) | 2015 (13.9) | 6.26 (4.53-8.65) |
| 8 | 718 (6.8) | 1165 (11.8) | 11.06 (7.00-17.47) | 351 (5.9) | 1532 (10.5) | 7.55 (5.42-10.52) |
| 9 | 368 (3.5) | 804 (8.1) | 14.90 (9.36-23.69) | 173 (2.9) | 999 (6.9) | 9.99 (7.04-14.17) |
| 10 | 190 (1.8) | 471 (4.8) | 16.90 (10.48-27.26) | 86 (1.4) | 575 (4.0) | 11.57 (7.88-16.99) |
| 11 | 98 (0.9) | 266 (2.7) | 18.51 (11.18-30.63) | 50 (0.8) | 314 (2.2) | 10.87 (7.06-16.71) |
| 12 | 30 (0.3) | 111 (1.1) | 25.23 (13.81-46.08) | 11 (0.2) | 130 (0.9) | 20.45 (10.26-40.73) |
| 13 | 12 (0.1) | 58 (0.6) | 32.95 (15.32-70.88) | 5 (0.1) | 65 (0.4) | 22.49 (8.60-58.80) |
| 14 | 3 (<0.1) | 11 (0.1) | 25.00 (6.46-96.70) | 3 (0.1) | 11 (0.1) | 6.34 (1.71-23.60) |
